# Explaining the Determinants of First Phase HIV Decay Dynamics through the Effects of Stage-dependent Drug Action

**DOI:** 10.1371/journal.pcbi.1002971

**Published:** 2013-03-28

**Authors:** James B. Gilmore, Anthony D. Kelleher, David A. Cooper, John M. Murray

**Affiliations:** 1 School of Mathematics and Statistics, University of New South Wales, Sydney, New South Wales, Australia; 2 The Climate Change Research Centre, University of New South Wales, Sydney, New South Wales, Australia; 3 The Kirby Institute, University of New South Wales, Sydney, New South Wales, Australia; 4 Centre for Applied Medical Research, St Vincent's Hospital, Darlinghurst, New South Wales, Australia; Imperial College London, United Kingdom

## Abstract

A recent investigation of the effect of different antiretroviral drug classes on first phase dynamics of HIV RNA plasma virus levels has indicated that drugs acting at stages closer to viral production, such as the integrase inhibitor raltegravir, can produce a steeper first phase decay slope that may not be due to drug efficacy. Moreover it was found that for most drug classes the first phase transitions from a faster (phase IA) to a slightly slower decay region (phase IB) before the start of the usual second phase. Neither of these effects has been explained to date. We use a mathematical model that incorporates the different stages of the HIV viral life cycle in CD4+ T cells: viral entry, reverse transcription, integration, and viral production, to investigate the intracellular HIV mechanisms responsible for these complex plasma virus decay dynamics. We find differences in the phase IA slope across drug classes arise from a higher death rate of cells when they enter the productively infected stage post-integration, with a half-life of approximately 8 hours in this stage, whereas cells in earlier stages of the infection cycle have half-lives similar to uninfected cells. This implies any immune clearance is predominantly limited to the productive infection stage. We also show that the slowing of phase IA to phase IB at day 2 to 4 of monotherapy, depending on drug class, is a result of new rounds of infection. The level at which this slowing occurs is a better indicator of drug efficacy than the slope of the initial decay.

## Author Summary

The infection of a cell by HIV proceeds through a series of stages and each stage can now be inhibited by an available antiretroviral drug class. It is known that different drug classes can result in different decay curves of plasma viral levels that are not well explained by current mathematical models of HIV dynamics. Here we develop a mathematical model that incorporates these stages of infection and show how it successfully reproduces plasma decay curves for the five classes of currently available antiretroviral drugs. Our modeling indicates that the efficacy of antiretroviral drugs is not solely described by the rate of decay of plasma viral levels as currently thought. Drugs such as the integrase inhibitor raltegravir will result in a faster initial decline of plasma viral levels compared to a drug that acts further from viral integration and production such as the CCR5 inhibitor maraviroc, even though they may have the same efficacy. Moreover, we find that infected cells only die at rates above the background level when they are in the productive phase, indicating that immune clearance is mostly absent from the early stages of HIV cellular infection. This is of particular concern given that most infected cells are in these early stages of infection.

## Introduction

Mathematical modeling of HIV infection has led to major advances in understanding HIV replication *in vivo* [1]–[4]. However, important questions still remain, and here we address how the underlying viral life cycle of HIV within CD4+ T cells can influence the first phase decay kinetics of HIV RNA plasma virus levels (pVLs) after the commencement of antiretroviral therapy (ART). We are motivated by results from trials with the integrase inhibitor (INI) raltegravir [5] which produces a more extensive first phase decay than antiretroviral drugs from other classes. More recent investigations with drugs from a number of classes indicate that first phase decay rates can differ between drug classes and may not be constant over the entirety of the first phase [6]. The impact of the stage at which inhibition occurs within the viral life cycle on first phase decay is currently poorly understood.

Early mathematical models estimated the underlying parameters of viral clearance and cell lifetimes from pVL dynamics [3], [7]–[10]. Some models incorporate time delays, and this has been shown to be particularly important in the modeling of HIV dynamics. Delays, which describe the amount of time required for HIV infection within a CD4+ T cell to progress from the stage inhibited by ART until viral production, are observed through an initial shoulder in pVL decay dynamics under ART [11]. Dixit *et al.* [12] showed that by incorporating delays, HIV dynamics were more accurately modeled, especially when drug efficacy is less than 100%, as is the case *in vivo* [13], [14]. The first phase decay rate has also been shown to be weakly dependent on the delay [15]. More complex time delay models have been introduced by Dixit and Perelson [16], who examined pharmacokinetics in a model with two delays, and Ouifki and Witten [17] who studied the stability of a three stage delay model.

The stage of the viral life cycle at which a drug acts has been incorporated into HIV mathematical models [18], [19], and stage-dependent inhibition has also been demonstrated *in vitro*. Sedaghat *et al.* [18] showed viral life cycle properties could impact pVL decline. In [20], Donahue *et al.* verified that drugs from separate antiretroviral classes will result in different times until virion production, where these differences depended on the stage of the HIV infection cycle being inhibited.

Recently, our group analyzed first phase dynamics during monotherapy with eight antiretroviral drugs [6]. Data were examined from early dose-ranging and viral dynamics studies of monotherapy with five drug classes: the INI raltegravir [5], the non-nucleoside reverse transcriptase inhibitor (NNRTI) rilpivirine [21], the nucleoside/nucleotide RTIs (NRTIs) abacavir and tenofovir [22], the entry inhibitors (EIs) enfuvirtide [23] and maraviroc [24], and the protease inhibitors (PIs) ritonavir [3], [25] and nelfinavir [26]. The time delay to initial pVL decay, when corrected for the pharmacokinetic delay, was found to increase as the inhibited replication stage is further from second generation viral export. These time delays were found to be about twice those observed for *in vitro* cell lines [27], indicating the process of viral replication occurs over a longer time period *in vivo* [6]. There was also evidence that the slope of the first phase becomes steeper for drugs with shorter time delays such as for the INI raltegravir. Moreover pVL decay was not constant over the course of what would be expected to be the first phase. The first phase of viral decay under monotherapy can be divided into two separate phases, IA and IB. Phase IA is the initial component of the first phase decay and lasts for 2–4 days. Phase IB follows and lasts until about day 10 with the phase IB decay being slower than the phase IA decay. Here we explore the biological determinants of these two phases in the context of stage-dependent modeling of drug inhibition.

We model the HIV life cycle in CD4+ T cells by incorporating the time required for HIV to progress between the major stages of the viral life cycle. We consider each of the stages in the cycle that can be impacted by ART as separate, discrete but interrelated entities. We describe how the HIV life cycle in CD4+ T cells influences viral decay dynamics after the commencement of ART and determine the conditions under which the phase IA decay kinetics depends on the stage of the HIV life cycle being inhibited. We find that different slopes of the first phase decay are to be expected for antiretroviral agents from separate classes, and this does not necessarily reflect variations in drug efficacy. Moreover we find that this detailed analysis, when linked to observations *in vivo* [6], indicates that the infected cell death rate only increases substantially above that for an uninfected cell at late stages of the infection cycle, belying any significant immune-mediated lysis during the pre-integration stages of infection.

## Materials and Methods

### Mathematical model

**Figure 1:**
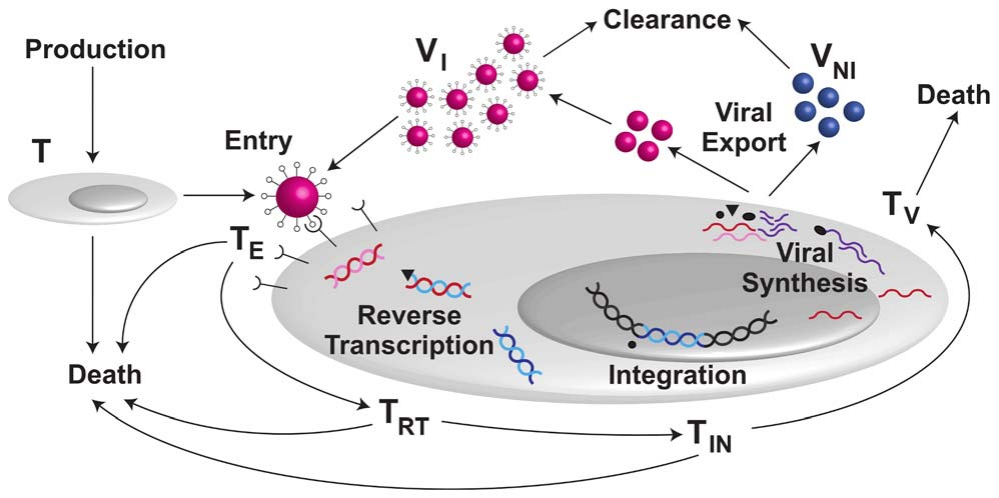
Model for HIV infection in CD4+ T cells. Production and loss includes CD4+ T cell production, CD4+ T cell death of infected and uninfected cells, and viral clearance. The major HIV infection stages in the model are: entry, reverse transcription, integration, and viral production. The model describes five types of CD4+ T cells: uninfected cells 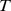, cells where HIV has passed entry 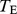, cells where HIV has passed RT 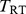, cells where HIV has passed IN 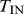, and productively infected CD4+ T cells 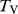. Virus comprises infectious virus 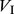, and non-infectious virus 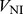 (when PIs are administered).

We mathematically model the major steps of the HIV life cycle as interlinked processes with explicit time delays. To accomplish this, the viral life cycle is split into four major stages: viral entry, RT, IN and virion production as shown in Figure 1. Our model focuses on the initial time delay and the period normally described as encompassing first phase viral decay after the commencement of monotherapy and therefore describes the viral life cycle in CD4+ T cells that are destined for productive infection. Latently infected CD4+ T cells and long-lived infected cells are not included in the model, as they are thought to contribute to the classic second phase that commences approximately 10 days after administration of ART [7].

When constructing the life cycle stages, we are guided by the current classes of antiretroviral drugs that inhibit infectious virion production (PI), entry (EI), reverse transcription (NRTI and NNRTI), and integration (INI), and for which delay estimates have been derived [6]. Although data are available for two different mechanisms of RT inhibition, we describe RT inhibition in a single stage to retain model simplicity and clarity. This means the model assumes RT occurs at the average of the measurements in Murray *et al.* [6] derived from analysis of decay kinetics following monotherapy with two NRTIs (abacavir and tenofovir) and one NNRTI (rilpivirine), which determined a 33 hour time period over which reverse transcription occurs. Consequently the modeled stage after this average RT time point but before integration includes cells that have not completed RT. These simplifications result in a four stage model that retains mathematical tractability, while also allowing an in-depth examination of the viral dynamics that result from stage-dependent inhibition.

The mathematical model is described by a system of seven first order differential equations, four of which are delay differential equations,
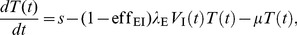
(1)

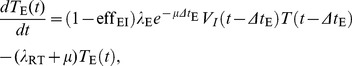
(2)

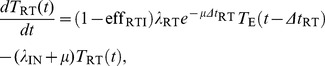
(3)

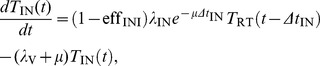
(4)

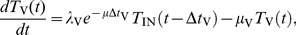
(5)

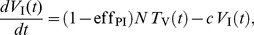
(6)

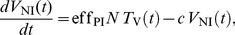
(7)where 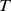 is the number of uninfected CD4+ T cells in the body at time 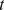 in days, and 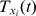 is the number of CD4+ T cells that have been infected by HIV that have passed the 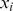 stage in the life cycle but have not reached the 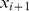 inhibitory stage. For example, 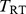 is the number of CD4+ T cells where HIV has passed the inhibitory effects of a RTI but where HIV DNA has not yet been integrated into the cell's genome. The mathematical model is represented schematically in Figure 1.

Virions can only be produced when a CD4+ T cell becomes productively infected denoted by the stage 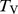. Virus is divided into two components describing infectious 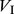 and non-infectious 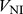 virus under the effects of a PI [3]. Although not all virions will be infectious even in the absence of a PI, we effectively incorporate this component into a lower infectivity 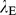, where 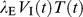 is the rate at which CD4+ T cells are infected, so that the non-infectious component under a PI is in addition to this background level. This is a simplification used by most authors modeling HIV dynamics, see for example [7], [15]. The factor of 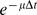 incorporates CD4+ T cell death during the time delay. When the CD4+ T cells are producing virions (as in Eq. 5), we allow a different death rate 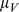, due to the immune response and/or cytopathic effects, compared to the death rate 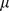 for the non-productive compartments. For perfectly efficacious drugs, this model can be solved exactly (see below).

Although our focus is monotherapy, the model does admit stable viral loads prior to therapy and also shows rapid increases in viral load when therapy is interrupted or in primary infection scenarios.We note the sum of all CD4+ T cells will only be constant prior to therapy. The relative CD4+ T cell proportions change after therapy commences with all infected stages decreasing and uninfected CD4+ T cells increasing. Viral levels are also constant prior to therapy and decrease thereafter.

Models that omit time delays allow some CD4+ T cells to progress from initial infection to viral production almost instantaneously. This is at odds with the time required for each of the biological mechanisms within the viral life cycle. To remedy this short-coming, each of the stages in our model incorporates its own time delay (see Eqs. 1–5). We have also linked the stages by the progression rates 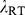, 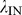, and 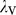, resulting in two parameters controlling the progression of infection at each stage of the viral life cycle. Having two parameters describing each stage allows us to essentially describe the mean and variance for the time taken between life cycle stages.

The parameters we use in the model are described in Table 1. We have chosen the parameters to broadly agree with the values used in the literature for similar models, see for example [3], [12]. We perform a sensitivity analysis on the model parameters to ensure our phase IA results are robust with regards to different parameter choices. As discussed later, phase IB is sensitive to most model parameters. The new parameters that we introduce to describe the life cycle are optimized to give good agreement with the monotherapy data we discuss later. Note that for the clearance rate, we take 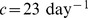 to agree with measurements derived from observations made during large volume plasma apheresis [9], although similar results are obtained with 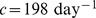 as estimated in Murray *et al.* [6].

**Table 1:** Summary of model parameters.

Parameter	Value	Units	Description	Source/Notes
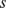	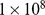	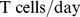	CD4+ T cell formation rate	Ref. [2], [18]
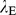	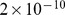	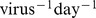	rate of entry	Ref. [16], [19]
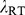	4.0	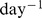	progression rate of RT	This work
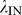	1.4	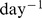	progression rate of IN	This work
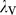	5.0	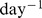	progression rate to viral budding	This work
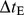	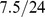	day	entry time delay	Ref. [6]
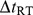	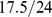	day	RT time delay	Ref. [6]
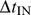	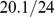	day	IN time delay	Ref. [6]
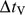	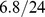	day	production time delay	Ref. [6]
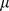	0.05	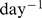	CD4+ T cell death rate	This work, Ref. [13], [16]
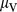	2.5	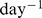	productive CD4+ T cell death rate	This work
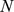	2000	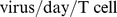	virion production rate	Ref. [16]
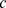	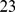	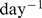	viral clearance rate	Ref. [9]
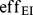	0.90–1.00		EI efficacy	
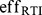	0.90–1.00		RTI efficacy	Monotherapy 0.90
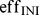	0.90–1.00		INI efficacy	Exact solution 1.00
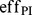	0.90–1.00		PI efficacy	

Parameters in the model were optimally chosen to match the monotherapy characteristics from Murray *et al.* [6], and 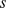, 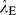, 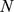, and 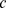 were chosen to be consistent with previous literature values.

In the presence of ART, four steps in the viral life cycle can potentially be inhibited. To incorporate this effect for RTIs and INIs, we have included 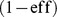 on the completion term for RT and IN in Eqs. 3 and 4, where 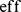 is the efficacy of the drug class in question. We assume abortive infection occurs for those T cells where the viral life cycle does not progress past the inhibition point of the drug class, see for example [28]. Alternatively, these cells can be returned to the uninfected CD4+ T cell pool and simulations under this assumption produced virtually identical results to those described here. For EIs, we assume the rate of entry/infectivity, determined by 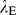, is simply reduced by a factor 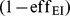 from the outset in Eqs. 1 and 2. The use of a PI inhibits maturation of virions and results in non-infectious virus, as modeled in Eqs. 6 and 7. Note that the time delays in the model do not explicitly depend on drug efficacy. We assume no pharmacokinetic delay in the simulations of each drug class so that time zero is when each drug commences inhibition of its stage in the HIV life cycle. The pharmacokinetic delay can be incorporated by an additional delay, see [16] for example, however this has already been estimated and subtracted in the analysis presented in [6].

### Analytical solution with perfect efficacy

When the efficacy of each monotherapy drug is perfect we can solve the system of equations analytically. For the case of INI monotherapy with perfect efficacy 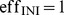, the solution is 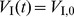 for 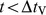, where 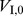 is the pVL at 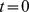 before ART and 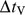 is the delay between HIV DNA integration and viral production, and for 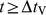 we have
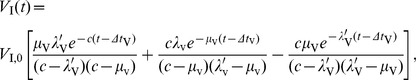
(8)where 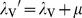. By examining the exponentials in Eq. 8, the rate of viral decay for an INI will be determined by 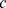, 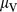, and 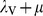. For RTI monotherapy with 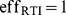 the solution is 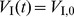 for 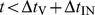, and for 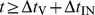 we have
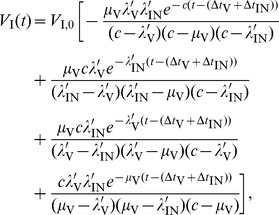
(9)where 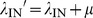. By examining the exponentials in Eq. 9, the viral decay for an RTI will be determined by 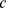, 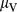, 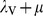, and 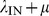. Compared to the INI, the presence of the rate of integration 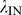, means the RTI viral decay can be different (and less) than the INI. The expressions for the two remaining inhibition classes, EIs and PIs, have five and six terms incorporating all previous rates and 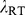 and 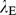 exponentiated respectively. These terms are analogous to those for the RTI and INI but with more complex four and five term denominators (not shown).

### Model data

The time delays between drug administration and effect on pVLs for the eight drugs from Murray *et al.* [6] are shown in Table 2. It should be noted that for complexity reasons our model does not differentiate between different drugs that act on the same stage of the viral life cycle. Since we represent only one RTI in the model, the time delay determined for that stage was taken as the mean of the initial time delays of rilpivirine, abacavir, and tenofovir. We use the maraviroc time delay for EIs since its estimates were based on more patient data than for enfuvirtide. The PI delay is averaged from the fits to nelfinavir and ritonavir data. For the INI we use the delay for the only integrase inhibitor for which data are available, raltegravir.

**Table 2:** Monotherapy data.

	Individuals	Delay (hours)	Slope ( 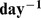 )	Std Dev. ( 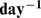 )
raltegravir	25	6.8	1.52	0.10
rilpivirine	36	11.4	0.93	0.42
abacavir	10	25.0	0.70	0.32
tenofovir	11	44.4		
enfuvirtide	7	32.2		
maraviroc	64	44.4	0.86	0.18
ritonavir	7	52.3		
nelfinavir	5	51.6	1.50	

Initial delay and phase IA monotherapy slopes when fitting biphasic decay curves separately to each drug group [6]. Standard deviations (Std Dev.) were obtained from the nonlinear mixed effects calculations for each drug. The number of patients used in the analysis is also shown. The entries that are empty under the slope category did not admit nonlinear mixed effect fits due to insufficient data. The pharmacological delay has been subtracted from the delays.

Determining the initial delays was the main purpose of the Murray *et al.* [6] investigation. To accomplish this, nonlinear mixed effects modeling of the pVL dynamics were constrained to a first phase slope with the same fixed effect across all drug classes. However, further analysis of first phase slopes for individual drugs indicated these could differ depending on the stage of the viral life cycle being inhibited. The first phase slopes from the latter analysis are shown in Table 2. Here we use the slopes of raltegravir, rilpivirine, and maraviroc for comparison with our model simulations using an INI, RTI, and EI respectively. No fitting of the phase IA slope for the PIs was employed given the small number of patients in the PI data. Although nonlinear mixed modeling indicated a decay rate of 1.5 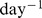 for nelfinavir, data on which this analysis was performed after the 2 day initial delay for PIs were only available at days 3, 6, and 9 leading to considerable uncertainty in this estimate. The estimated slope for nelfinavir was inconsistent with the trend of all patient data.

### Model optimization

The longitudinal analysis of Murray *et al.* [6], clearly identified two parameters relevant for the phase I dynamics of drugs acting at different inhibition stages administered in monotherapy. These parameters, the initial time delay until viral load reduction and the phase IA decay slope, imply that two parameters can be used to constrain the model at each replication stage [4], [7]. This guided our construction of the stage dependant model as discussed previously. Since our model does not predict the time delays from first principles, we fix the observed time delays to those in [6] (given in Table 2).

To fit the remaining phase IA slopes from the monotherapy data, we assume an efficacy of 90% for each drug (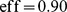), and optimize over five parameters 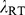, 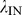, 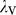, 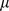, and 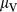. These five parameters control the phase IA slopes, see Eqs. 8 and 9. As noted in Murray *et al.* [6], pVL decreased by more than 90% for each drug class indicating an efficacy of at least 90%. We note that we have five parameters and only three phase IA slopes meaning we can only formally constrain three parameters. However, given the model consists of a series of interconnected replication processes, the slope of later acting drugs (such as the EI) are not independent of the earlier replication processes (such as integration and viral production). This means we are able to obtain reasonable bounds on the parameters, except 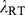 as discussed below. The remaining model parameters not listed above do not explicitly control the phase IA slopes of the various drug classes but influence phase IB. We fix these other parameters to those given in Table 1. Note that the time delays in the model are corrected for the average half-life of progression in each stage 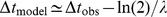. For the model optimization calculation we construct a chi-squared measure by comparing the modeled and observed phase IA slopes 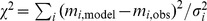, where we sum over the drugs with reliable non-linear mixed effects phase IA slopes and 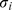 is the estimated standard deviation of the observed slopes (Table 2). We combine the RTI errors via Monte Carlo to give 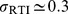. Only the phase IA slope of the viral decline is used to constrain the model, since it is largely independent of efficacy for 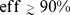. Phase IB is sensitive to efficacy and other model parameters and is not used to constrain the model.

To estimate the parameters that control the phase IA slope for the various drug classes, we use a five parameter optimization and grid search in 0.1 intervals from 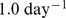 to 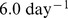 in 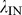, 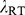, 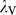, and 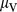. For 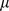 we use 0.05 intervals from 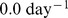 to 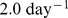. The optimal values for phase IA are given in Table 1. To estimate the errors on the five parameters, the 95% confidence intervals are obtained by using a bootstrap method, randomly dropping 20% of data points for each drug and then re-running the optimization procedure. We also tested dropping 20% of patients with similar results. The error on each parameter was estimated with the other parameters held fixed at their optimal values. This gives at 95% confidence; 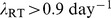, 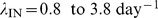, 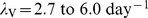, 
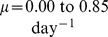
, and 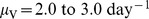. One parameter 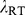 is only bounded below at 95% confidence due to there being only three non-linear mixed effects slopes. The best estimates for these parameters are provided in Table 1.

Matlab Version 2010b (The MathWorks Inc, Natick MA, USA) and Mathematica 8.0 (Wolfram Research, Champaign IL, USA) were used to solve the mathematical model and fit the monotherapy data.

## Results

### Conditions leading to different decay slopes

Our aim here is to show that by taking into account the stages inhibited by drugs, pVL can exhibit different first phase decay rates by drug class that are not simply determined by differing drug efficacy. As a consequence, even if the drugs have perfect efficacy (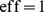) their slopes will differ. Under the assumption of perfect drug efficacy we can solve the system of differential equations Eqs. 1–7 analytically which provides useful parameter bounds for the numerical computations. We presented the solutions for the INI and RTI in Eqs. 8 and 9 respectively.

Analysis of the perfect efficacy solutions for the INI and RTI viral load decays shows how the progression, death, and clearance rates can potentially determine the phase IA slope. For an INI with perfect efficacy 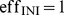, phase IA decay is determined by the rates 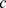, 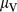, and 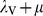 (Eq. 8). Since the viral clearance rate 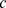 is much larger than any other progression or death rate parameter, it will not play a role in determining the phase IA slope. For the INI then, the phase IA slope will be given by the smallest of 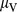 and 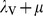. Similarly when perfect efficacy RTI monotherapy is applied 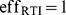, the phase IA slope is determined by the three rates 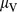, 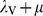, and 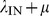 (Eq. 9).

**Figure 2:**
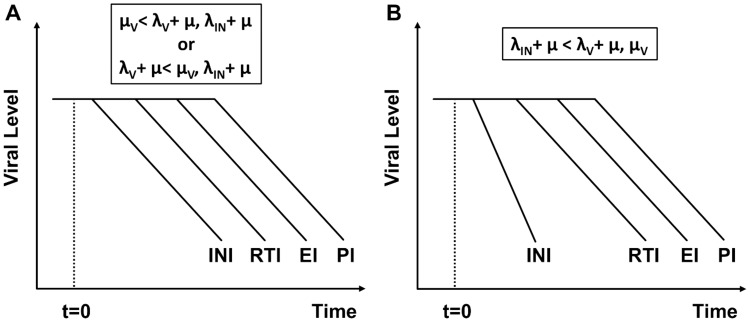
Illustration of the different phase IA slope possibilities in the model. (A) When the productively infected death rate is low, or the rate to viral production is low, a difference in phase IA slopes is not seen between the drug classes. (B) When the progression rate to IN complete is low compared to the productively infected death rate or the rate to viral production, a phase IA slope difference will be observed. In this case the INI will have a steeper decay than the RTI, EI, or PI.

Comparison of the parameters controlling the phase IA decay show how the decay can be different between an INI and RTI. When the rates obey the inequalities 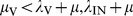 or 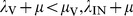, the phase IA decay rate with an RTI is no different to that with an INI. However when 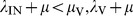 the phase IA decay rate for an INI will be larger than for an RTI. Hence phase IA decay slopes will differ for an RTI and an INI under the conditions that
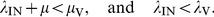
(10)Note that if the death rate of cells in the early stages of infection 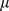, is equal to 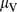, a phase IA slope difference will not be observed – implying 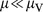 if a large difference in INI and RTI decay slopes is observed. Similar bounds can be derived if decay rates differ with the other drug classes. We have illustrated these inequalities in Figure 2, which shows that when the variance of movement from RT complete to IN complete is large, i.e. 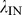 is small and 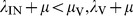 is true, the INI decay is steeper than the RTI, EI, and PI slope.

### Comparison to observed monotherapy pVL decay

**Figure 3:**
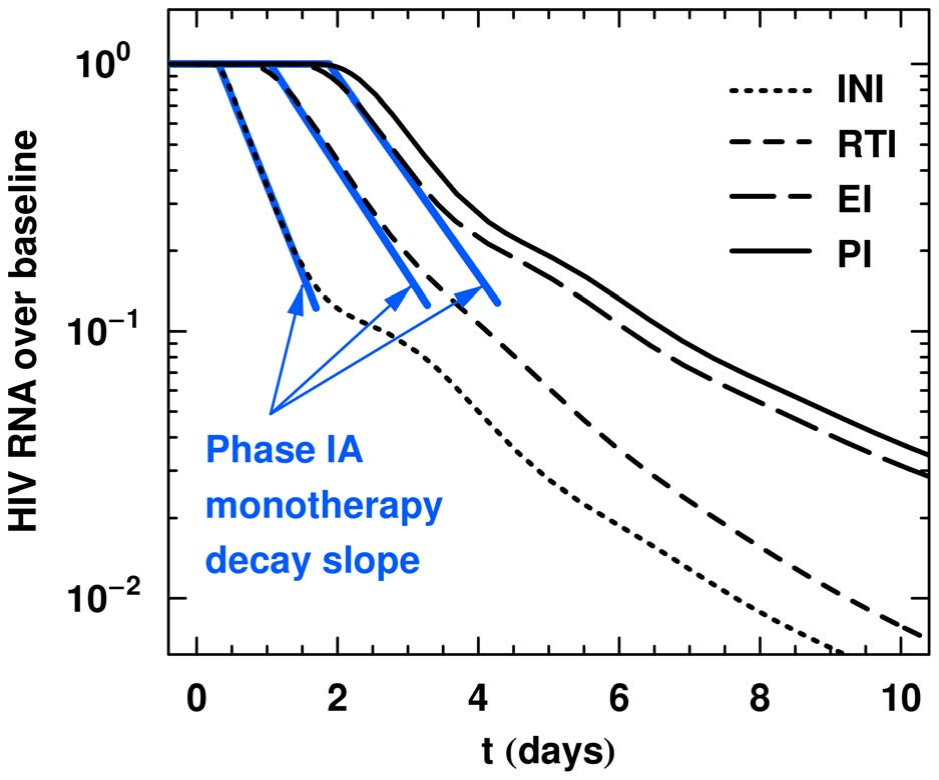
Phase IA and time delay comparison. Comparison of time delays and phase IA decay slopes from the data in Table 2 and the model with parameters given by Table 1, with model simulations of monotherapy commencing at time 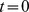. The y-axis shows virus levels normalized by baseline value at 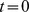. The dotted, short dashed, long dashed, and solid lines are the model simulations in the presence of an INI, RTI, EI, or PI respectively. *This convention is used in all subsequent figures*. The indicated gray lines display the mean phase IA decay rates for the INI, RTI, and EI commencing after the initial delay for each drug (Table 2, [6]). No PI slope is shown due to insufficient data. Efficacy is set to 0.90 for each drug class.

**Figure 4:**
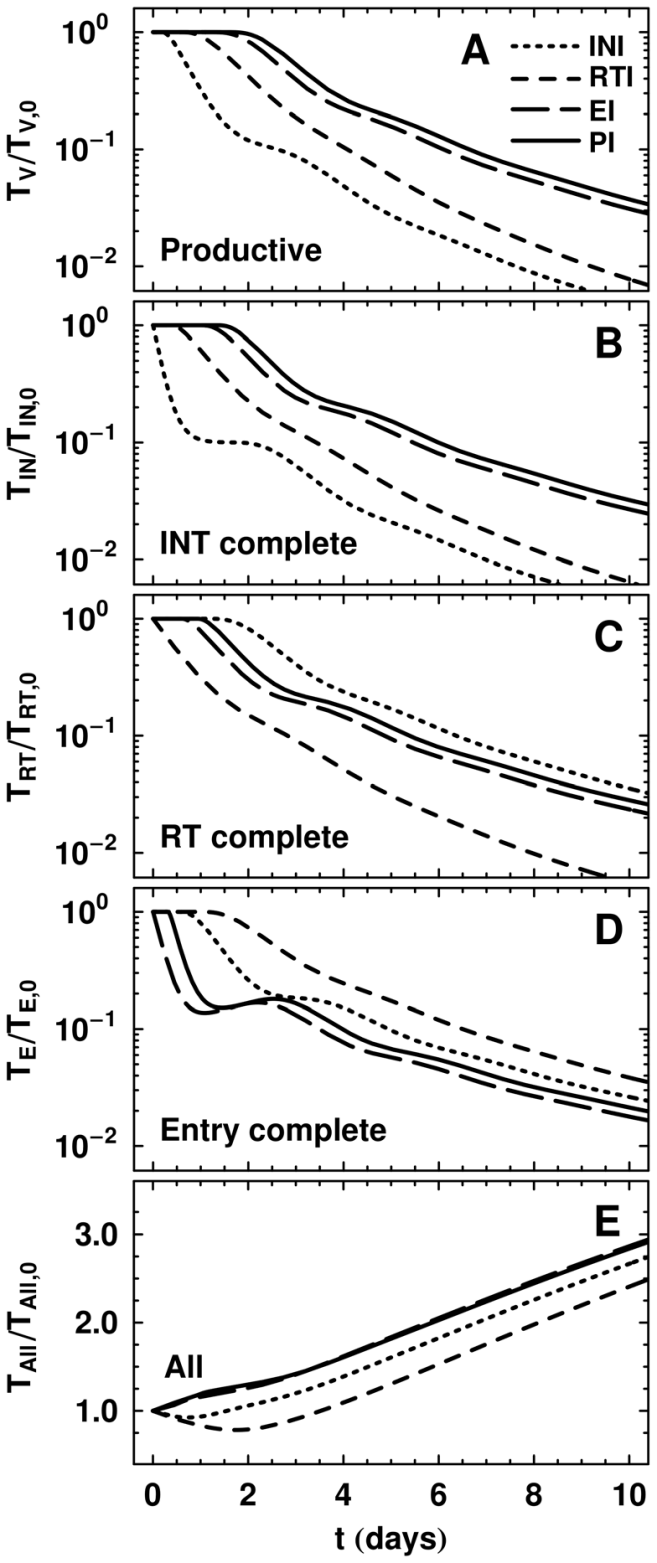
Companion CD4+ T cell dynamics to **Figure 3** for the optimal model. Here monotherapy is administered for the four different drug classes. All T cell species have been normalized to 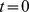 and are denoted generically by 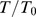. (A) Productive CD4+ T cells. (B) CD4+ T cells with integration complete. (C) CD4+ T cells with reserve transcription complete. (D) CD4+ T cells with entry complete. (E) All CD4+ T cells irrespective of their infection stage, including uninfected CD4+ T cells. Comparison of (A) to Figure 3 shows viral load tracks the number of CD4+ T cells producing virus 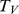 regardless of drug class, with the remaining infected CD4+ T cell species dynamics depending on the stage of inhibition. The final panel (E) shows the sum of all CD4+ T cells in the model 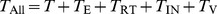. Note that the target plus infected CD4+ T cells 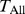 increase quasi-linearly as anticipated after the removal of the majority of infection with ART. Efficacy is 0.9.

**Figure 5:**
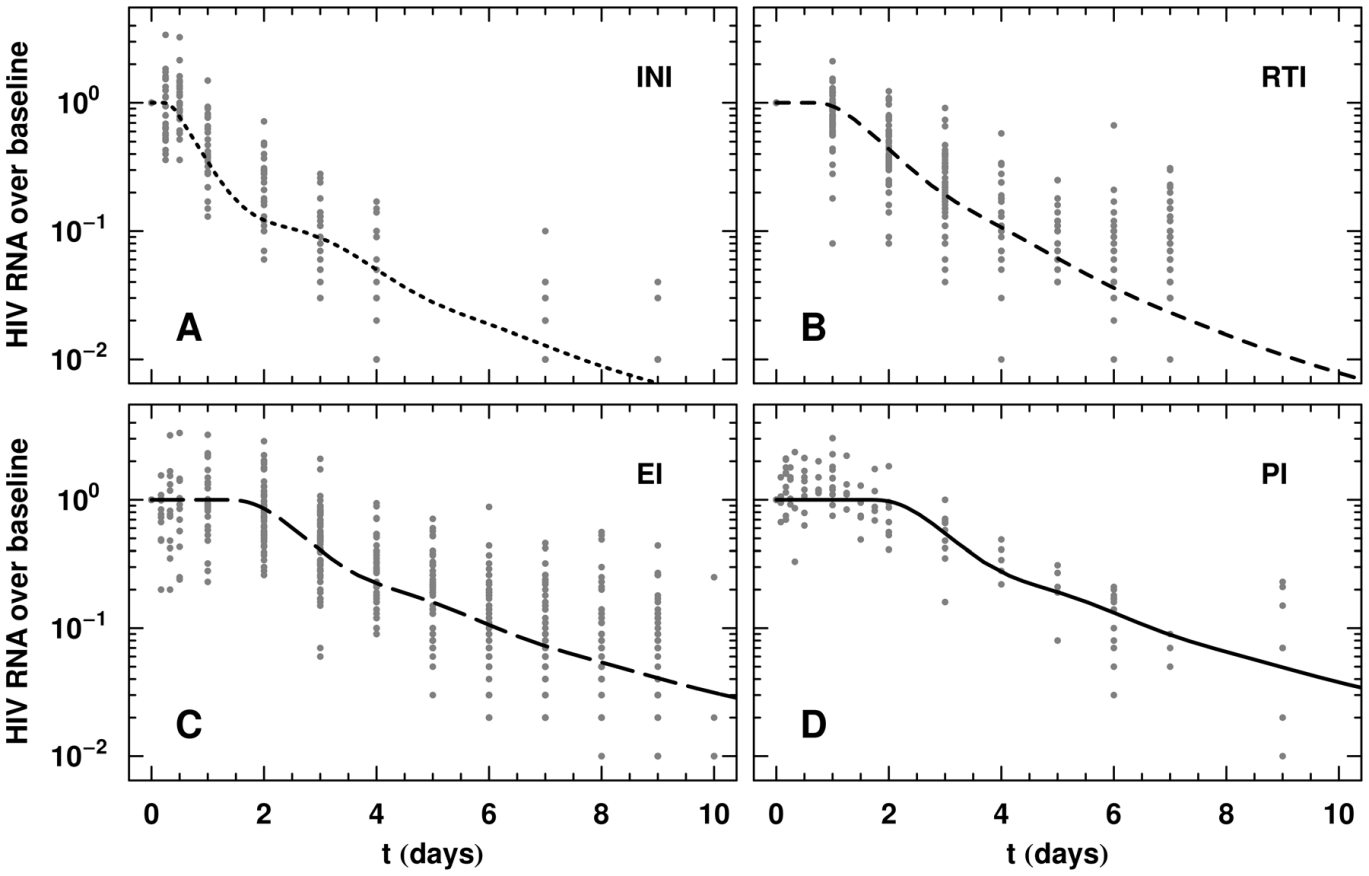
Qualitative comparison with the raw plasma viral load data. The raw plasma viral load data from [6] and the model with parameters given in Table 1 have been overlayed. The complete monotherapy data sets are shown. (A) For the INI, we show viral load data during raltegravir treatment. (B) For the RTI, rilpivirine, abacavir, and tenofovir are shown. (C) For the EI, enfuvirtide and maraviroc are shown. (D) For the PI, ritonavir and nelfinavir are shown. Good agreement is seen across the four drug classes in the model. We note the model is fitted to the longitudinal analysis of the assay data from [6]. Efficacy is set to 0.90 for each drug class.

To reproduce the measured phase IA decay rates for the four drug classes, the analysis above indicates the inequalities in Eq. 10 must hold and the death rate of productively infected CD4+ T cells 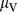, must be substantially larger than the death rate 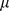 for cells that have not yet reached the stage of productive infection 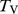 at perfect efficacy. Numerical solutions of the model with more realistic drug efficacies of 90%, and optimally chosen parameters, capture both the initial delays and the steeper phase IA decay slopes as the drug class acts closer to viral production (Figure 3). The distinct phase IA slopes in Figure 3, particularly for the INI, as well as the later phase IB profiles were all produced with the same 90% efficacy for each drug simulated as monotherapy. The optimal values for phase IA are given in Table 1. Simulations for each of the uninfected and infected stages of CD4+ T cells, 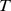 and 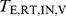 are shown in Figure 4. We also overlay the pVL data and the model (Figure 5) with the parameters from Table 1. Good agreement is seen across the drug classes with the optimally chosen model parameters.

### The death rate of productively infected CD4+ T cells

To reproduce the faster phase IA decay for the INI, we found the death rate of productively infected CD4+ T cells 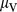, must be substantially larger than the death rate 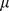 for cells that have not yet reached the stage of productive infection 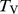. From our model, a productively infected death rate of 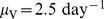 allows us to model the steep phase IA decay of the INI, as shown in Figure 3. Based on our analysis we also found that the death rate for cells undergoing infection but which are not in the productive 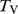 stage must be low with 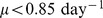, and smaller values of 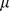 than this bound produced better fits to the data. The upper bound of 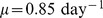, is considerably lower than the range of values for 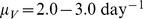. We also investigated the case where the death rate could differ by stage of infection rather than be fixed at the one value 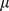. However this did not produce substantially better fits and still required these death rates by stage to be less than 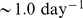 as above.

**Figure 6:**
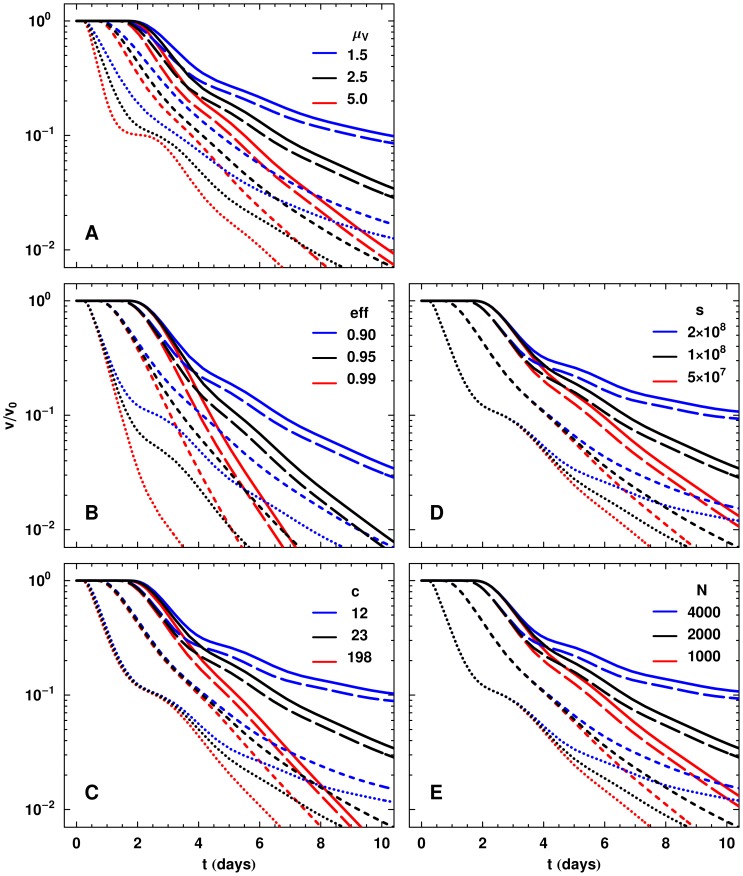
Comparison of pVL dynamics when individual model parameters are altered. Parameters are modified from those in Table 1, while all other parameters are held constant. (A) From this panel we can see that the productively infected cell death rate 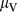 can control the slope in phase IA and the relative difference between phase IA slopes. (B) As efficacy 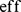 is changed, the phase IA slope is not strongly altered. However, the length of phase IA and the start of phase IB at 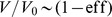 are dependent on 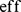. (C) Modifying the viral clearance rate 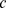 changes the initial delay slightly, but does not substantially modify phase IA. After the transition to phase IB, 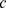 influences the decay considerably. (D) The production rate of uninfected CD4+ T cells 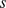, and (E) virion production rate per productively infected CD4+ T cell 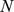. Both 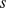 and 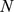 do not modify the time delays or phase IA, but affect phase IB. Efficacy is set to 0.90 in (A) and (C)–(E).

Simulations with the productively infected cell death rate set to 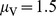, 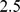, and 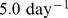 verify the influence that a high death rate in this stage can have on pVL dynamics (Figure 6A). We find that for 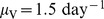, which is still greater than the death rate estimated for productively infected CD4+ T cells in the literature of 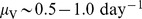 [1], [3], [7], [25] (where intracellular infection stages have not been included), the slope of phase IA does not appreciably differ between the drug classes, and does not duplicate the fast decay produced by the INI raltegravir. On the other hand, the larger values 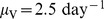, and 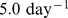 result in the fast decay achieved with an INI but also produce the slower rates of decay with other drug classes.

This behavior with large values for the productively infected death rate 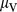 is predicted by the analytically-derived solution under the assumption of complete efficacy for the individual drugs. As discussed for the exact solution, we find that as the productively infected death rate 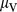 increases above the sum of the death and the progression rate of the previous infection stages, 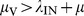, then the phase IA slopes become significantly steeper for the INI compared to an RTI and the other drug classes. The optimal parameter values for the model assuming non-perfect efficacy of 90% of 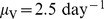 and 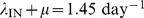, are consistent with this relationship.

### Variability around the average time delay

Although the mean time delay between stages of infection is mostly determined by the values 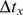, the progression rates 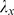 impact on the variability around this mean; high progression rates lead to all cells progressing from one stage to the next at almost the same time 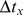; low progression rates lead to some cells progressing slowly and others quickly, a much more heterogeneous process. The sharpness of the transition after the time delay to phase IA is controlled by the progression rates 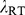, 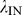, and 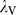 in a similar manner. For example in Figure 3, we see the transition is particularly sharp for the INI, whereas for the PI, a more extensive shoulder is observed. Since the effect is cumulative with respect to the progression rates, the INI produces a sharp transition since it only depends on 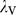, whereas the EI and PI transition to the phase IA decay will depend on the size of 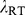, 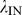, and 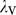.

**Figure 7:**
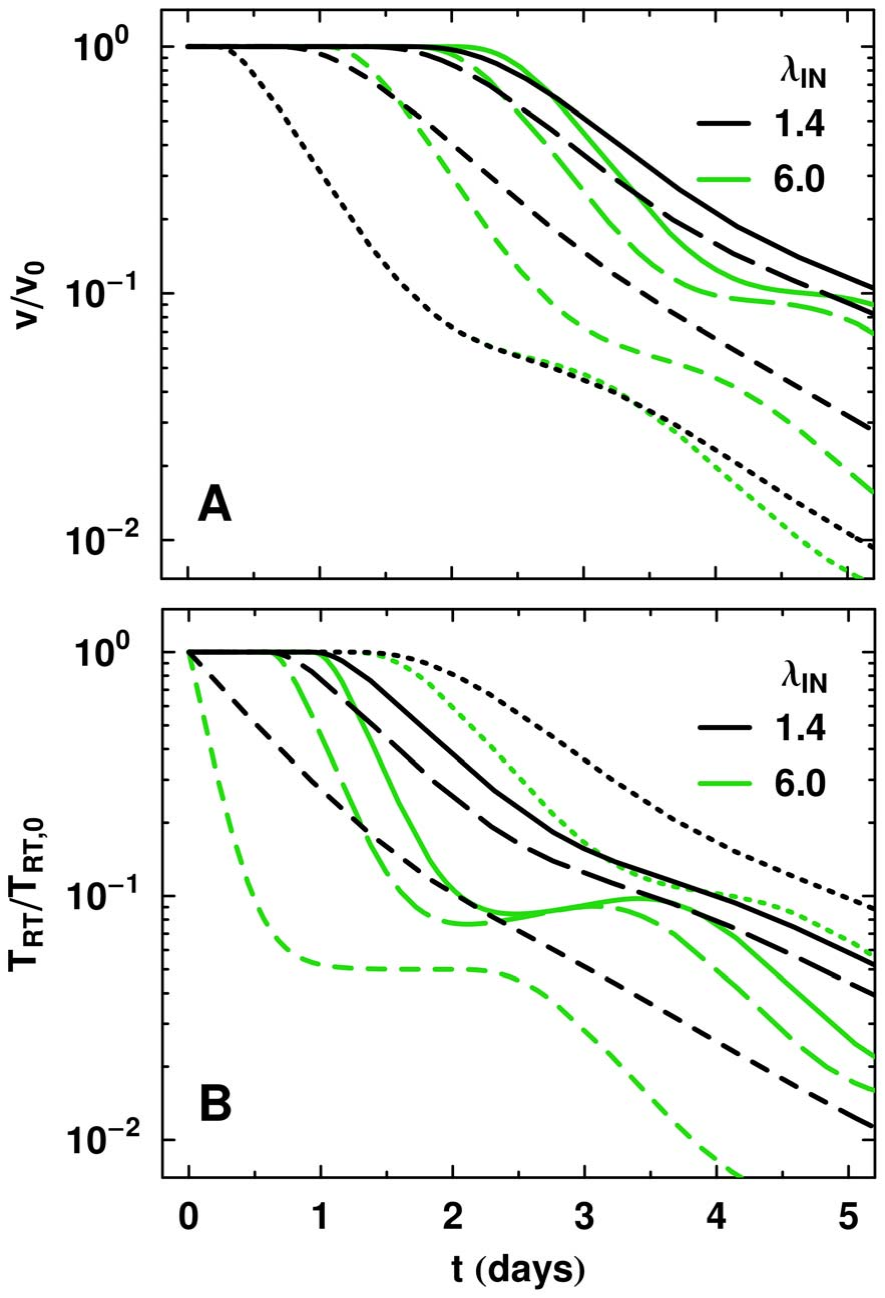
Influence of the progression rate to IN, 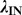
 **, on the pVL curves.** (A) A relatively low rate of 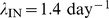 (black curves) giving a variance of 0.5 days around the mean time to IN 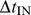, leads to a large slope difference between the INI and RTI, compared to a fast rate 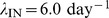 (gray curves) with standard deviation of 0.1 days around the mean for virus progressing from the middle of RT to IN. (B) In this panel, we show how the CD4+ T cells move from RT to IN for all drug classes. For 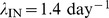, note the relatively slow rate at which the 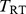 progress from the RT stage and move to IN, is reflected in the slow decrease in 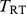. An efficacy of 0.95 is used for each drug.

Slow progression rates for 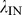 (Figure 7A) and 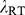 (not shown), or large variability about the mean time, lead to large phase IA slope differences between drug classes acting on either side of the respective stage in the life cycle. Our exact solution to the model with complete efficacy also predicted this behavior. This is observed in the monotherapy data of [6] which indicated 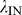 is small, rather than 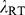. For completeness we also plot in Figure 7B 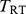 relative to baseline, showing the long slow decline of the 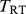 curve resulting from small values 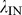. As our RT stage was set at the average time for when reverse transcription occurs over a 33 hour time period, and 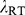 and 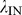 describe the rates leading up to and progressing from this mid-point, the low values of these rates are consistent with the slow completion of RT and/or IN.

### Drug class efficacy and the first phase

Although drug efficacy will modify the phase IA slope at low levels, once above a certain efficacy of 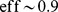, the phase IA slope is determined by other effects, such as the decay rate of productively infected CD4+ T cells as described above. Sensitivity of phase IA and IB decay to different efficacy rates are shown in Figure 6B. At higher efficacy, we observe a lengthening of the phase IA decay, but no change in the decay rate, and the damping of any other effects due to the time delays in the decay curves.

Drug efficacy can significantly modify phase IB, the slower part of the traditional first phase decay, as observed by Murray *et al.* [6] and has also been commented on by other investigators [25], [29]. The phase IB decay rates depend on all model parameters but the relative pVL at which this phase occurs is dependent on drug efficacy, as well as drug class. The pVL relative to baseline is approximately 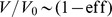 so that greater efficacy results in a more substantial decrease before the commencement of phase IB (Figure 6B). These results indicate that phase IB originates from new rounds of infection that progress despite the presence of the drug.

### Virion clearance rate

The virion clearance rate in the model was fixed at 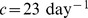 [9]. To determine if 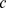 had any appreciable effect on the phase IA dynamics, we examined different values of the clearance rate, 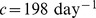 as determined by [6], and a smaller value 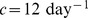. As seen in Figure 6C, the value of 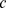 has little effect on the total time delay associated with each drug class and produces no appreciable modification of phase IA. The virion clearance rate 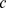 controls the length of time circulating virus is available to infect CD4+ T cells through the infection term 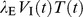. This means that changes in 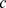 will modify phase IB in the pVL curves (Figure 6C).

### Target cell production

The availability of target cells was determined by the parameter 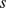. Its value had no effect on the delay or phase IA slope (Figure 6D). However, as the production rate of target cells 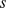 is increased, there was a more prominent slowing of the phase IB decay at later times. This indicates that the availability of target cells to infection plays an important role in phase IB, and further implies that phase IB results from new rounds of infection by circulating virions. We note that increasing the number 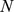 of virions produced has a similar effect to increasing 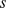 (Figure 6E).

## Discussion

With the increasing number of antiretroviral drug classes, monotherapy and other trials provide data that allows examination of the HIV life cycle in greater detail than ever before. A recent study of monotherapy trials by Murray *et al.* [6], demonstrated a systematic trend in the observed time delays of pVL curves – as the drug class acts further from viral export, the initial delay increases. To examine the effect of the HIV replication cycle on pVL curves and first phase dynamics, we created a mathematical model explicitly incorporating stages of the life cycle of HIV in CD4+ T cells.

**Figure 8:**
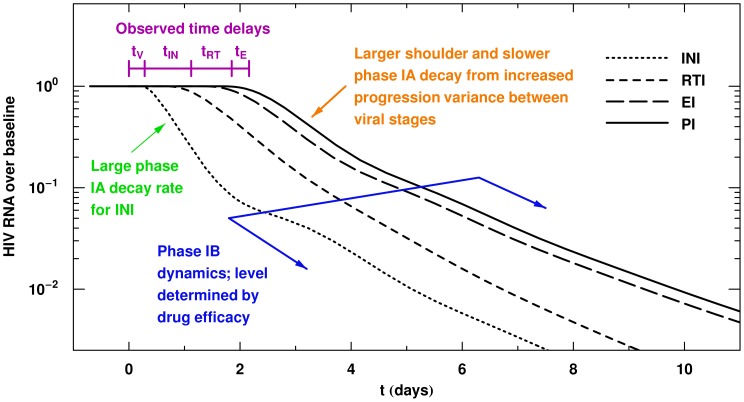
Plasma HIV RNA curves for the four drug classes INI, RTI, EI, and PI. The dotted, short dashed, long dashed, and solid lines are model simulations where the effects of an INI, RTI, EI, and PI respectively were modeled. The key features of the model have been labeled. The time delays are given by 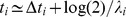. An efficacy of 0.95 has been used for each drug.

We found that with stage-dependent drug action, the traditional first phase which arises from productively infected CD4+ T cells, has three components: 1) an initial delay preceding any decrease in the pVL curves after commencing monotherapy, 2) a steep decay at the end of this shoulder, which we call phase IA, and 3) a slower decay, called phase IB, that starts at day 2–4. The precise characteristics of these three features depends on where the drug intervenes in the viral life cycle, and on the mean and variance of the time required to progress between different stages of infection (determined by parameters 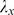 and 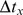). The observed initial delay describes the time from a given stage in the viral life cycle until virion export. The slope of phase IA is determined by the drug class/classes being used, the death rate 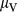 of productively infected cells and the progression rates between viral life cycle stages: 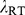, 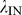, 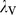. The length of phase IA is largely determined by drug efficacy but is also influenced by the drug class/classes being used (Figure 6B). Phase IB is sensitive to most parameters in the model, since it is a result of new rounds of infection. Plasma virus levels are substantially lower at initiation of phase IB at larger drug efficacy and phase IB pVLs remain higher with greater target cell availability. Figure 8 summarizes the main features observed from the data and duplicated by our model.

One of the main motivations for investigating a stage-dependent model of HIV decay kinetics was to understand the observed differences in phase IA slopes between INIs and RTIs, EIs, and PIs. Specifically, what mechanisms lead to INIs having steeper phase IA pVL decays? Nelson *et al.* [15] showed a time delay can change the slope of phase IA by 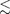 20% depending on the efficacy; however, this cannot account for the 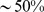 faster decay for raltegravir compared to rilpivirine (Table 2). In our analysis we determined a large slope difference is achievable, but only under certain conditions. These are: (1) there is a substantial variance around the mean in the commencement times for the stage that includes part of RT plus IN of HIV DNA, with the progression rate being on the order of the mean time 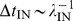, (2) the death rate of productively infected CD4+ T cells must be high at a rate of approximately 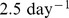, (3) after HIV DNA integration all cells that are to become productively infected without an intervening latent stage will do so quickly with a small variance (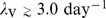), (4) the viral clearance rate 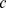 is also high, and (5) the death rates 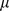 of the infected but unproductive stages are low and specifically must satisfy 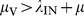. Under our mathematical modeling, all of these conditions must hold for a large difference in slopes to be observed as described in Murray *et al.* [6]. Given these conditions are independent of specific antiretroviral effects, they inform the biology of the interaction between HIV-1, CD4+ T cells, and the immune system.

A large death rate of productively infected cells 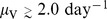 (half life of 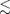 8 hours), was required to model the monotherapy pVL decay data. Previous values in the literature estimated a productively infected CD4+ T cell death rate of approximately 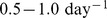 [1], [3], [7], [25]. These estimates mostly did not include a directly calculated initial delay, nor did they discriminate between the two parts of the first phase, phase IA and IB, that have now been observed by a number of investigators [6], [25], [29]. Furthermore no consideration was given to the drug class/classes being used, which we have seen to considerably influence the rate of decay over the first 10 days of ART (Figure 3).

Our models shows that the slope of phase IA is not simply determined by the death rate of productively infected cells 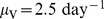, but is also determined by the rate at which cells progress through the stages of infection. For example, for an INI we must also account for the rate at which integrated CD4+ T cells become productively infected at the rate 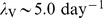. The phase IA slope is then approximately given by 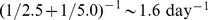, consistent with the observed phase IA INI slope (Table 2). This means movement through the entire replication cycle must be considered when estimating phase IA slopes.

We determined the productively infected stage of an infected cell has a half-life of 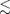 8 hours with average duration of 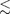 12 hours. In [6] the total HIV life cycle was determined to be 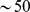 hours. This implies there are approximately 3-fold more cells in the early stages of infection than actually producing virions, and suggests that viral proteins such as *tat* produced soon after integration, are produced relatively late in the total viral infection cycle. Of the infected cells that are not yet productive, about 2/3 are in the RT stage.

Both the numerical and analytical solutions indicated that the death rate of cells in the early stages of infection 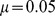 (95% confidence interval of 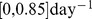), is much lower than the death rate during the productively infected stage 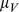. The value 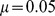 corresponds to an average lifespan of 20 days for these cells and is hence not much different to the lifespan of uninfected target cells which are most likely in an activated state. *In vitro* analysis has indicated that CD8+ T cells can recognize gag-derived epitopes within the first 2 hours of SIV infection of primary CD4+ T cell lines and are capable of eliminating these cells early in the infection cycle [30]. Our *in vivo* analysis on the other hand, indicates early immune recognition of CD4+ T cellular infection is compromised, possibly due to loss of HIV-specific CD4+ T cell help at primary infection [31], or that immune clearance is abrogated by persistent viremia [32]. This suggests that *in vivo* expression of cytotoxic T cell epitopes by infected CD4+ T cells only occurs efficiently once viral proteins are made *de novo* from the integrated provirus. This is consistent with the current understanding of the processing and presentation of antigens through the MHC-class I pathway [33]. This delay may be critical for viral control and may explain the delays that compromise the effectiveness of CD8+ T cell cytotoxic responses in controlling the virus infection [34]. Further this delay may be exacerbated by Nef-induced down-regulation of MHC-I and CD4, which tends to make these cells more immunological silent to cytotoxic T cells and therefore more difficult to clear [35]. This delay in immune clearance of infected cells may be due to viral processes blocking pathways leading to apoptosis. The expression of Nef proteins early in the infection cycle and intercellular contact with macrophages has been observed to reduce apoptosis of cells proceeding to infection [36]. Our modeling cannot determine the processes behind the large clearance rate at the productively infected stage so that either cytopathic effects of virus production or immune-mediated destruction may be responsible.

Further, our analysis also reveals that relative drug efficacy may be measured very early, through the length of phase IA. This may prove useful in the early stages of drug development. Our mathematical model incorporating the stages of HIV infection has proved instrumental in explaining a number of as yet unexplained features from pVL curves under different antiretroviral drug classes.
